# Changes over time in Lithuanian schoolchildren’s attitudes toward addictive behaviors: Promoting and preventing factors

**DOI:** 10.1371/journal.pone.0208481

**Published:** 2018-12-05

**Authors:** Darius Leskauskas, Virginija Adomaitienė, Cornelis A. J. De Jong, Birutė Vorevičiūtė, Rima Juknaitė

**Affiliations:** 1 Department of Psychiatry, Lithuanian University of Health Sciences, Kaunas, Lithuania; 2 Behavioral Science Institute Radboud University, Nijmegen Institute for Scientist Practitioners in Addiction, Nijmegen, Netherlands; Cardiff University, UNITED KINGDOM

## Abstract

**Background:**

Concern is growing about the high prevalence of traditional and new forms of addictive behaviors among young people due to the health risks and a better understanding of the factors causing these behaviors is needed.

**Aim:**

To evaluate tendencies in the attitudes of Lithuanian schoolchildren toward addictive behaviors over a three year period and to ascertain the promoting and preventing factors of such behaviors.

**Methods:**

The researchers developed a survey which was conducted twice over a three year period. The sample consisted of pupils in the 5^th^, 9^th^ and 12^th^ grades (N = 1590, age range 11–19 years) from both urban and rural areas.

**Results:**

Both the recognition of and involvement in addictive behaviors significantly increased with age. Motivation to abstain due to internal factors decreased with age and increased among pupils already involved in addictive behaviors. Time- and age-related differences were found regarding substance abuse and behavioral addictions. Whilst betting adverts were increasingly noticed over time, smoking adverts were decreasingly noticed over the three year period and it was concomitant with inconsistent changes in self-reported involvement in these behaviors.

**Conclusions:**

Most significant changes in the attitudes of Lithuanian pupils toward addictive behaviors occur between the ages of 11 and 15 years. However, age-related changes differ for the pupils’ attitudes toward substance abuse and behavioral addictions. Increasing awareness of the potential risk of addictive behaviors does not prevent their increasing prevalence with age. Increased risk of involvement in addictive behavior correlates with decreased internal motivation to abstain from addictive behavior and decreased recognition of its potential risks. No clear correlation was found between significant changes in noticing adverts and involvement in addictive behaviors.

## Introduction

Alcohol, tobacco and gambling are legal and potentially highly addictive, and they all have an impact on the social determinants of health (e.g., poverty, social inequality), pose common risks to both physical (e.g., injury, disease) and mental health (e.g., suicide, addictions), and can lead to criminal behavior (e.g., violence, drunk driving) [1]. Smoking remains the single greatest preventable cause of mortality worldwide, being a major risk factor for a number of life-threatening diseases, including various cancers and cardio-vascular and lung diseases [2,3]. Adolescence is the developmental period when involvement in addictive behavior and the risks for its health-disturbing consequences are most likely to occur [4,5,6]. Relatively new forms of addictive behaviors such as computer gaming, internet addiction and betting are primarily affecting young people and there is growing concern among the research and health policy fields due to the risk factors for health problems [7,8,9,10,11]. These forms of addictions, termed “behavioral addictions”, are now being introduced into the main classification systems for psychiatric disorders [12]. The prevalence of addictive behaviors is high among adolescents in Lithuania. Results from the European School Survey Project on Alcohol and Other Drugs (ESPAD) showed that alcohol and cannabis usage rates among Lithuanian pupils exceeded the European average [13]. However, preventative measures can only be effective if they both reach the target population and also influence the factors facilitating development of these addictive behaviors [14,15]. Studies have shown that being exposed to people drinking and smoking and seeing advertisements involving alcohol and tobacco are key risk factors for not only starting drinking and smoking at a young age, but also having a higher consumption of these substances and being at increased harm from them [16,17,18].

The results of our 2010 survey showed the prevalence of potentially addictive behaviors and acknowledgement of their risks increasing with the age of the adolescent participants. The television and Internet were the sources of information most often mentioned by respondents as influencing their wish to try as well as to abstain from trying addictive behaviors [19].

Our current study aimed to gain knowledge that might help to develop effective prevention programs. Between the 2010 and 2013 surveys, some prevention programs were implemented, such as restriction of the public advertizing of tobacco products and the school based program “Empty it 2012–2013”. In this program pupils were involved in the development of prevention projects focusing on addictive behaviors. However most schools continued with traditional prevention lessons developed by teachers, police officers or health care specialists.

The aim of the study was to evaluate tendencies in the attitudes of Lithuanian schoolchildren toward addictive behaviors over a three year period and to ascertain the promoting and preventing factors of such behaviors. The main research questions were: What are the attitudes of the adolescent schoolchildren (target population of prevention programs) towards addictive behaviors? What factors do they consider as influencing their adoption of addictive behaviors or abstaining from them? How do these attitudes and factors change with age? Do these attitudes and factors change significantly over the three year period?

## Materials and methods

### Procedure

This study evaluated attitudes of 11–19 year-old pupils toward addictive behaviors over a three year period. The model of our study was a mixture of a cohort study and a repeated cross-sectional study. Two surveys were conducted in the same 6 schools with a 3 year period interval and applying the same anonymous self-report questionnaire.

### Participants

A total of 1590 schoolchildren were polled in the study (age range 10–19 years, 51.8% female and 48.2% male) from six schools in different towns in Lithuania, with 60.5% and 39.5% from urban and rural areas respectively, thus giving a representative sample of Lithuanian schoolchildren of early, middle and late adolescent age. The first survey involved only those in the 5^th^, 9^th^ and 12^th^ grades, and the second survey involved those in the 5^th^, 8^th^ and 12^th^ grades. The survey was conducted twice in all schools in October, 2010 (n_1_ = 856) and October, 2013 (n_2_ = 734). Pupils who were in the 5^th^, 9^th^ grades in year 2010 were inquired twice with a 3 year interval; other pupils (12^th^ graders in 2010 and 5^th^ graders in 2013) were inquired once. Sub-samples of the surveys did not differ significantly in terms of distribution according to grade, gender or place of residence.

### Instruments

The survey used was developed by the researchers based on their previous experience, background literature and the study’s research questions. Self-reported data received with anonymous questionnaire can be considered reliable when studying addictions related personal attitudes and behaviors of adolescents [20,21]. Questionnaire used to collect the data consisted of an introduction and 12 multiple-choice questions separated into three sections: 1) demographic data; 2) questions about attitudes toward addictive behaviors and factors promoting or preventing them, such as: a) Do you think this could be an addictive behavior?, b) In your opinion, what behavior is most intensively advertised on television, in magazines or on the Internet?, c) What makes you abstain from trying or repeating an addictive behavior?; 3) questions about involvement in addictive behaviors.

A detailed description of the questions used was provided in a previous publication about this study along with the results of the first survey [19]. The introduction provided information about the study’s aims, the anonymous nature of the answers and the fact that participation was entirely voluntary. The following definition of addictive behavior was provided: “Addictive behavior is frequently repeated, at least once a day or week and it is behavior that is potentially harmful to health, learning, working life, relationships with friends or family or to any other area of life”.

### Statistical analysis

Time-related analysis was conducted by comparing two different groups—children of the same age surveyed after a three year interval. Age-related analysis was conducted by comparing different age-groups (5^th^ vs 8^th^/9^th^ vs 12^th^ grade) within the sub-samples of the surveys and within two follow-up groups—the same children interviewed after a three year interval. Within-group comparisons were conducted between 5^th^ grade pupils in 2010 who were 8^th^ grade pupils in 2013 (they constitute group A, n = 280) and 9^th^ grade pupils in 2010 who were 12^th^ grade pupils in 2013 (they constitute group B, n = 238). Statistical analysis was conducted applying within-group comparisons. Within-person analysis could not be done due to confidentiality issues involved. Pearson’s chi-squared test for categorical data was used to calculate the difference in distribution of the attitude-related variables between the sub-groups, in terms of the school grade and the year of the survey. IBM SPSS Statistics Version 21.0. was used to analyze the data.

### Ethical considerations

The study was given ethical approval by the Lithuanian University of Health Science’s Biomedical Research Ethics Committee (No. BEC-LSMU-379) and each of the participating schools’ administrations. Informed consent procedure was carried out in accordance with the recommendations of Ethics Committee. Informed consent of the parents/guardians of the pupils was obtained from the schools committees of the parents’ representatives. Verbal informed consent of the pupils was obtained after providing them with verbal and written information about the study. Information was provided about the study, anonymity of the answers and the free choice to disagree to participate. Those who agreed to participate were asked to complete and return the anonymous questionnaires. Questionnaires were collected and analyzed by the researchers with no access for the teachers or school administrations.

## Results

Table 1 provides descriptive data on the attitudes of pupils about what they considered to be addictive behaviors, which of these behaviors were most noticeably advertised and which of these behaviors they were involved in. Comparisons made within the surveys revealed some repetitive patterns of age-related changes in the attitudes of pupils toward addictive behaviors over the three year period. Recognition that behavior can be addictive and potentially harmful significantly increased with age for all forms of addictive behavior in both surveys. For traditional or substance abuse [19] addictive behaviors—smoking, use of illegal drugs, consumption of strong and weak alcohol—increase of recognition was most significant between the 5^th^ and 8^th^/9^th^ grades. For relatively newer forms of addictive behavior in Lithuania (computer gaming, betting), a significant increase in recognition was observed between the 8^th^/9^th^ and 12^th^ grades. Smoking, use of illegal drugs and strong alcohol consumption were most frequently and almost equally as often recognized as potentially addictive. The potentially addictive nature of weak alcohol (beer, cider) consumption, computer gaming and betting was recognized significantly less frequently (Table 1).

**Table 1:** Descriptive statistics and comparison within the surveys of the pupils’ attitudes toward which behaviors are addictive, their involvement in such behavior and noticing advertising relating to that behavior.

	Survey 2010 n_1_ = 856				Survey 2013 n_2_ = 734			
	5^th^ grade n = 280	9^th^ grade n = 291	12^th^ grade n = 285	Difference between the grades	5^th^ grade n = 182	8^th^ grade n = 314	12^th^ grade n = 238	Difference between the grades
Consider behavior as potentially addictive								
Consumption of weak alcohol	58.6%	69.0%	73.7%	χ^2^ = 17.0 p<0.01	63.9%	70.8%	75.3%	χ^2^ = 8.5 p<0.1
Consumption of strong alcohol	64.2%	86.1%	91.2%	χ^2^ = 87.4 p<0.01	68.3%	85.4%	90.4%	χ^2^ = 51.2 p<0.01
Smoking	68.0%	89.7%	90.2%	χ^2^ = 80.9 p<0.01	73.9%	88.3%	93.8%	χ^2^ = 48.9 p<0.01
Use of illegal drugs	62.4%	88.2%	91.9%	χ^2^ = 110.9 p<0.01	72.8%	85.7%	93.3%	χ^2^ = 47.5 p<0.01
Computer gaming	53.4%	54.9%	62.9%	χ^2^ = 9.1 p = 0.05	48.9%	52.7%	66.9%	χ^2^ = 23.6 p<0.01
Betting	45.9%	51.6%	68.9%	χ^2^ = 37.9 p<0.01	52.2%	55.2%	69.0%	χ^2^ = 17.4 p<0.01
Self-reported involvement in addictive behavior								
Consumption of weak alcohol	5.0%	33.3%	34.7%	χ^2^ = 90.7 p<0.01	2.8%	21.0%	31.4%	χ^2^ = 56.6 p<0.01
Consumption of strong alcohol	0.7%	15.1%	20.0%	χ^2^ = 55.7 p<0.01	2.2%	13.7%	23.8%	χ^2^ = 44.4 p<0.01
Smoking	2.5%	20.3%	25.6%	χ^2^ = 63.4 p<0.01	1.7%	14.6%	25.1%	χ^2^ = 44.7 p<0.01
Use of illegal drugs	0	4.1%	3.9%	χ^2^ = 12.7 p<0.01	0.6%	5.1%	5.0%	χ^2^ = 8.3 p<0.05
Computer gaming	39.6%	29.2%	14.7%	χ^2^ = 45.3 p<0.01	18.9%	21.3%	19.2%	χ^2^ = 1.5 p>0.1
Betting	6.1%	5.5%	2.8%	χ^2^ = 4.8 p>0.1	1.1%	6.7%	6.7%	χ^2^ = 9.5 p<0.05
Noticed as one of the most intensively advertised addictive behavior								
Consumption of weak alcohol	58.3%	68.3%	63.3%	χ^2^ = 6.0 p<0.05	58.7%	60.3%	69.0%	χ^2^ = 6.1 p<0.05
Consumption of strong alcohol	68.8%	72.1%	62.1%	χ^2^ = 6.7 p<0.05	62.0%	67.9%	59.8%	χ^2^ = 4.2 p>0.1
Smoking	39.5%	49.0%	40.0%	χ^2^ = 6.6 p<0.05	38.0%	34.3%	26.4%	χ^2^ = 7.0 p<0.05
Use of illegal drugs	14.1%	10.0%	14.0%	χ^2^ = 2.8 p>0.1	19.0%	15.6%	6.3%	χ^2^ = 16.6 p<0.01
Computer gaming	20.7%	17.6%	23.9%	χ^2^ = 3.4 p>0.1	16.2%	14.0%	17.6%	χ^2^ = 1.3 p>0.1
Betting	20.7%	17.9%	14.0%	χ^2^ = 4.3 p>0.1	32.4%	37.5%	40.6%	χ^2^ = 2.9 p>0.1

An increase in age-related self-reported involvement in addictive behaviors was observed in both surveys for most forms of such behavior (Table 1). The most significant increase in involvement in almost all forms of addictive behavior was observed between the 5^th^ and 8^th^/9^th^ grades. The only exception was computer gaming, involvement in which decreased significantly with age in the 2010 survey and remained at an equal level in all age-groups in the 2013 survey. Computer gaming and consumption of weak alcohol were the most frequently self-reported addictive behaviors in all age-groups in both surveys, followed by smoking and consumption of strong alcohol.

The results of the within-group comparisons emphasize the importance of change between the 5^th^ and 8^th^ grades (Table 2). There was a significant increase in the recognition of and involvement in all types of traditional or substance abuse addictive behavior in group A (p<0.01 for all differences in the distribution of within-group compared variables). In group B we found a significant increase only in self-reported involvement in strong alcohol consumption (χ^2^ = 6,54; p = 0,03). The dynamic in behavioral addictions was different—a significant decrease in self-reported involvement in computer gaming was found in group A (χ^2^ = 23,6; p<0.001), whilst a significant increase in recognition of computer gaming as an addictive behavior was found in group B (χ^2^ = 7,9; p = 0,01). A significant increase in recognition of betting as an addictive behavior was found in both groups which was concomitant with increased noticing of betting advertising (p<0.01 for all differences in the distribution of within-group compared variables) (Table 2).

**Table 2:** Comparison of changes in noticing advertising, recognizing potentially addictive behaviors and getting involved in addictive behaviors within the sub-groups.

Addictive behavior	Changes in noticing advertising		Changes in recognizing as addictive		Changes in self-reported involvement in addictive behavior	
	Sub-group A n = 280	Sub-group B n = 238	Sub-group A n = 280	Sub-group B n = 238	Sub-group A n = 280	Sub-group B n = 238
Consumption of weak alcohol	χ^2^ = 0.2 p = 0.6	χ^2^ = 0.1 p = 0.8	χ^2^ = 9.89^▲^ p<0.001	χ^2^ = 2.90 p = 0.2	χ^2^ = 34.73^▲^ p<0.001	χ^2^ = 0.28 p = 0.8
Consumption of strong alcohol	χ^2^ = 0.6 p = 0.8	χ^2^ = 8.8^▼^ p<0.01	χ^2^ = 43.69^▲^ p<0.001	χ^2^ = 2.38 p = 0.3	χ^2^ = 37.41^▲^ p<0.001	χ^2^ = 6.54^▲^ p = 0.03
Smoking	χ^2^ = 1.7 p = 0.2	χ^2^ = 28.2^▼^ p<0.01	χ^2^ = 42.52^▲^ p<0.001	χ^2^ = 2.79 p = 0.2	χ^2^ = 28.62^▲^ p<0.001	χ^2^ = 1.7 p = 0.4
Use of illegal drugs	χ^2^ = 0.2 p = 0.6	χ^2^ = 2.4 p = 0.1	χ^2^ = 44.97^▲^ p<0.001	χ^2^ = 7.3^▲^ p = 0.02	χ^2^ = 15.9^▲^ p<0.001	χ^2^ = 0.2 p = 0.8
Computer gaming	χ^2^ = 4.6^▼^ p = 0.03	χ^2^ = 0.1 p = 0.9	χ^2^ = 0.05 p = 0.9	χ^2^ = 7.9^▲^ p = 0.01	χ^2^ = 23.6^▼^ p<0.001	χ^2^ = 7.34 p<0.1
Betting	χ^2^ = 19.9^▲^ p<0.01	χ^2^ = 33.2^▲^ p<0.01	χ^2^ = 5.51^▲^ p = 0.06	χ^2^ = 17.2^▲^ p<0.001	χ^2^ = 1.12 p = 0.5	χ^2^ = 0.3 p = 0.8

^▲^ statistically significant increase (p<0.05),

^▼^ statistically significant decrease (p<0.05)

In the two surveys, the most prevalent addictive behaviors—consumption of weak and strong alcohol and smoking—were also most frequently noticed in advertising. However, age-related changes were different in both surveys and in the within-group comparisons. In the 2010 survey there was a significant increase in noticing the advertising of both smoking and strong and weak alcohol in the 9^th^ grade and a decrease in the 12^th^ grade (Table 1). In the 2013 survey there was a significant decrease in noticing the advertising of strong alcohol in the 12^th^ grade and advertising of smoking in the 8^th^ and 12^th^ grades (Table 1). Within-group comparisons revealed that a significant decrease in noticing strong alcohol advertising in group B was associated with an increased self-reported involvement in its consumption (Table 2). There was a decrease in noticing computer gaming adverts in group A and smoking adverts in group B which was not associated with any changes in the involvement in these behaviors. Finally, a significant increase in noticing betting adverts in both groups was associated with an increase in the recognition of this behavior as addictive but not with changes in involvement in it.

The survey results and within-group comparisons show similar tendencies in the age-related changes of the participants’ attitudes toward the factors motivating their abstinence from addictive behaviors (Table 3). Fear of health impairment and to ruin one’s life were most often mentioned as motivating abstinence, but the impact of these fears decreased with age. The importance of personal beliefs as determinants of internal control significantly decreased in the 8^th^/9^th^ grade but increased in the 12^th^ grade. The importance of fear of parents as an external control factor increased in the 8^th^/9^th^ grade and decreased in the 12^th^ grade. The importance of other external factors such as fear of the police, financial cost and availability of substances increased from 5^th^ to older grades, however changes between the 8^th^/9^th^ and 12^th^ grades differed only slightly in both surveys (Table 3). The within-group comparisons confirm the same age-related dynamics in the two groups. In group A the importance of fear of health impairment, fear to ruin one’s life and personal beliefs decreased significantly with age, whilst the importance of financial cost and availability increased significantly. In group B the importance of fear of health impairment, fear to ruin one’s life and fear of parents decreased significantly with age, while the importance of personal beliefs and financial cost increased significantly (Table 3).

**Table 3:** Comparison of age-related changes toward the factors mentioned as motivating abstinence from addictive behaviors in the 2010 and 2013 surveys.

Factor mentioned as motivating abstinence	Survey 2010 n_1_ = 856			Survey 2013 n_2_ = 734			Changes between the grades in the two surveys			Change within group A	Change within group B
	5^th^ grade %	9^th^ grade %	12^th^ grade %	5^th^ grade %	8^th^ grade %	12^th^ grade %	5^th^ grade	8^th^,9^th^ grade	12^th^ grade		
Fear of health impairment	78.1	74.9	70.1	76.7	64.4	63.6	χ^2^ = 0.1	χ^2^ = 7.8**	χ^2^ = 2.4	χ^2^ = 13.4**	χ^2^ = 7.9**
Fear to ruin one’s life	79.9	69.4	65.1	72.2	61.6	54.8	χ^2^ = 3.6*	χ^2^ = 4.0*	χ^2^ = 5.8*	χ^2^ = 23.7**	χ^2^ = 11.9**
Personal beliefs	47.7	31.6	47.2	44.4	38.7	41.4	χ^2^ = 0.4	χ^2^ = 3.3	χ^2^ = 1.7	χ^2^ = 4.8*	χ^2^ = 5.4*
Fear of parents	34.4	44.0	23.9	27.2	38.1	20.5	χ^2^ = 2.6	χ^2^ = 2.1	χ^2^ = 0.8	χ^2^ = 0.89	χ^2^ = 32.5**
Fear of police	20.8	22.0	12.7	15.6	16.2	21.3	χ^2^ = 1.9	χ^2^ = 3.3	χ^2^ = 7.0**	χ^2^ = 2.0	χ^2^ = 0.03
Too expensive	10.4	38.5	34.5	16.1	34.0	47.7	χ^2^ = 3.2	χ^2^ = 1.3	χ^2^ = 9.3**	χ^2^ = 46.5**	χ^2^ = 4.5*
Not available	9.0	7.9	13.0	8.9	14.6	7.1	χ^2^ = 0.01	χ^2^ = 6.7**	χ^2^ = 4.9*	χ^2^ = 4.4*	χ^2^ = 0.1

* p<0.05,

** p<0.01

In both surveys, pupils already involved in any kind of addictive behavior more often than not-involved mentioned that external control factors motivate them to abstain from addictive behaviors: fear of parents (χ^2^ = 16,6; p<0,001) and police (χ^2^ = 41,6; p<0,001) or financial cost (χ^2^ = 36,9; p<0,001). Pupils abstaining from addictive behaviors were motivated significantly more frequently by internal control factors such as personal beliefs (χ^2^ = 37,5; p<0,001), fear of life (χ^2^ = 7,0; p = 0,03) or health impairment (χ^2^ = 8,23; p = 0,02).

Comparing between the groups showed a significant increase in noticing betting advertising in all age-groups in the 2013 survey (χ^2^ = 7.9 for 5^th^ grade, χ^2^ = 28.5 for 8^th^/9^th^ grade, χ^2^ = 47.4 for 12^th^ grade, p<0,01 for all groups). This change was associated with different changes in self-reported involvement in this behavior in different age-groups: a significant decrease in the 5^th^ grade (χ^2^ = 6.9, p = 0.03) and close to significant increase in the 12^th^ grade (χ^2^ = 4.7, p = 0.09). An increase in noticing weak alcohol advertising in the 8^th^ grade (χ^2^ = 4.1, p = 0.04) was associated with a significant decrease in self-reported involvement in this behavior in the same age-group (χ^2^ = 12.1, p<0.01). A decrease in noticing smoking advertising in the 8^th^/9^th^ grades (χ^2^ = 13.4, p<0.01) and 12^th^ grades (χ^2^ = 10.8, p<0.01) was not concomitant with any change in self-reported involvement in this behavior.

## Discussion

The results of our study show some stable patterns in the attitudes of pupils toward addictive behaviors and the changes in their attitudes over the years. Understanding the potential risks of addictive behaviors increases with age but it does not prevent increased involvement in those behaviors. This tendency can be influenced by the observed change in motivation—the risk increases when internal motivation to abstain (fear for life and health impairment and personal beliefs) decreases. Early and middle adolescence seems crucial in this respect as most significant changes in the attitudes towards involvement in addictive behaviors and motivation to abstain were found in this period. A similar pattern was reported by Salas-Wright et al. who found significant differences in disapproval of addictive behavior (cannabis use) between the age groups of 12–14 and 15–17 [22]. These observations point to the need to develop prevention programs involving young adolescents by focusing on strengthening their internal motivation to abstain from addictive behaviors and increasing their awareness of the risks of behavioral addictions. Such a prevention strategy is supported by the results of a recently published study with a similar design of representative sample cohort follow-up showing that the increase in risk perception of cannabis use was related to decreased cannabis use after a 2 year period in the same mid-adolescence cohort [23]. An overview of 40 year results of a national survey on drug use in adolescents conclude that perceived risk had served as a reliable predictor of trends in the use of a number of substances [24], a finding recently confirmed the relationship between substance abuse risk perception and use in German population [25].

Lithuanian pupils do appear to improve over time in their understanding of the risks of traditional addictive behaviors such as substance abuse but they less frequently recognize the risks of relatively newer forms of behavioral addictions, often related to computer and Internet use. This seems to show the need to improve our understanding and management of the multidimensional digital media that influence adolescents’ addictive behaviors including both substance abuse and behavioral addictions [26,27].

The significant increase in noticing betting advertising in all age-groups in the 2013 survey was probably due to the fact that betting advertising significantly increased during sport events in Lithuania since 2010. However, no clear evidence was found that indicated that these changes in advertising of addictive behaviors influence the pupils’ behavior—increased noticing of betting advertising and decreased noticing of smoking advertising over the three year period were followed by no or inconsistent changes in the involvement in these behaviors. This may be due to limitations of the study such as the rather short interval between the surveys and perhaps insufficient sample size. It highlights the need for studies with a longer duration and designed as repetitive surveys of bigger nationally representative cohort samples [20]. The fact that the study involved a representative sample and included both substance abuse and behavioral addictions can be considered strengths of the study.

## Conclusions

Results of the three year follow-up study show that the most significant changes in the attitudes of Lithuanian pupils toward addictive behaviors occur between the 5^th^ and 9^th^ grades corresponding to pupils between 11 and 15 years of age. Age-related changes differ for attitudes toward addictive behaviors of substance abuse and behavioral addictions. Increasing awareness of the potential risk of addictive behaviors doesn’t prevent their increasing prevalence with age. Increased risk of involvement in addictive behavior correlates with both decreased internal motivation to abstain from addictive behavior and decreased recognition of the potential risks. Fear for health impairment and fear to ruin one’s life were the most frequent motives to abstain but their impact decreased with age. Significant changes in noticing advertising of addictive behaviors were found over 3 year period however no clear correlation was found with the changes in the self-reported involvement in such behaviors. This shows the need to develop age-specific and behavior-specific prevention programs and to evaluate their effectiveness in controlled trials with bigger samples of pupils.
